# Evaluation of tumor targets selected from public genomic databases for imaging of pancreatic ductal adenocarcinoma

**DOI:** 10.1038/s41598-025-00517-1

**Published:** 2025-05-16

**Authors:** Nada Badr, Luca Ten Elshof, Ruben D. Houvast, Lysanne D. A. N. de Muynck, A. Stijn L. P. Crobach, Gerard J. P. van Westen, Ronald L. P. van Vlierberghe, J. Sven D. Mieog, Alexander L. Vahrmeijer, Peter J. K. Kuppen

**Affiliations:** 1 Department of Surgery, Leiden University Medical Center, Leiden, The Netherlands; 2 Department of Pathology, Leiden University Medical Center, Leiden, The Netherlands; 3 Drug Discovery and Safety, Leiden Academic Centre of Drug Research, Leiden, The Netherlands

**Keywords:** Pancreatic cancer, Tumor-target selection, Fluorescence-guided surgery, Data-driven approach, Molecular imaging, Cancer imaging, Tumour biomarkers, Target identification

## Abstract

Pancreatic ductal adenocarcinoma (PDAC) is an aggressive malignancy with a 5-year survival rate of approximately 5–7%, and complete surgical resection remains the only curative treatment but is often unfeasible. Fluorescence-guided surgery (FGS) using tumor-targeted probes may improve tumor visualization and facilitate complete resection. This study aimed to identify and validate tumor targets for FGS during PDAC resection procedures. RNA expression data from over 4000 cell surface genes, obtained from public genomic databases, were analyzed to identify genes encoding PDAC-associated proteins. Eleven potential tumor targets were identified, including CEACAM5, TMPRSS4, COL17A1, CLDN18, and AQP5. Protein expression was evaluated by immunohistochemistry (IHC) in tissues from 44 PDAC and 7 chronic pancreatitis (CP) patients. All targets, except COL17A1, showed significantly higher expression in PDAC tissue compared to healthy pancreatic, CP, and duodenal tissue (*p* < 0.001), as well as in tumor-positive versus tumor-negative lymph nodes. Especially CEACAM5, TMPRSS4, and AQP5 were identified as the most promising targets for distinguishing PDAC from healthy tissues and detecting lymph node metastasis during FGS. The development of probes targeting multiple markers, such as AQP5 with CEACAM5 and/or TMPRSS4, may help overcome interpatient variability and enhance detection across patients.

**Supplementary Information:**

The online version contains supplementary material available at 10.1038/s41598-025-00517-1.

## Introduction

Pancreatic ductal adenocarcinoma (PDAC) accounts for 90% of pancreatic cancers and is known for its poor clinical prognosis with a 5-year survival rate of around 5–7%^1^. Surgery with pre-operative chemo(radio)therapy is the only potentially curative treatment approach. However, survival remains poor, and local recurrence rates are high, mainly due to incomplete resections (R1). Complete resections (R0) have been linked to improved patient survival^2^. R1 resections are often caused by inadequate tumor localization during surgery^3^. Existing preoperative imaging methods may present limitations in accurately distinguishing between healthy tissue and vital tumor tissue, chronic pancreatitis (CP), or fibrosis^3,4^. Consequently, approximately 7% of pancreatic surgeries are performed unnecessarily for benign diseases^5^. In addition, the effects of neoadjuvant therapy (NAT) further complicate adequate tumor recognition before and during surgery. Preoperative imaging techniques may, moreover, fail to detect small metastatic lesions^6^. Lastly, intraoperative assessment of tumor margins through visual inspection, palpation, or imaging techniques is difficult due to the complex spread pattern of PDAC and the presence of inflammatory tissue^7^.

Real-time fluorescence-guided surgery (FGS) allows surgeons to accurately visualize and fully remove tumors. Additionally, FGS assists surgeons in identifying metastatic lymph nodes during the procedure, which is crucial, as the removal of metastatic lymph nodes is associated with improved patient survival^1^. Indocyanine green (ICG) is an example of a clinically approved non-targeted near-infrared (NIR) dye that accumulates in areas with increased blood flow such as tumors or inflamed tissue. However, its effectiveness in pancreatic cancer is limited due to the lack of an enhanced permeability and retention effect, a consequence of the highly stromal nature of PDAC^8^. Tumor-targeted probes consisting of a targeting moiety conjugated to a NIR fluorescent dye have been proposed for real-time FGS in PDAC and their feasibility has been demonstrated^9^. Favorable properties of a target include an extracellular location, and homogeneous overexpression in tumor tissue and metastases compared to normal surrounding (inflammatory) tissue^10^.

Some previously developed tracers for pancreatic cancer have targeted CEACAM5^11^, EGFR^12^, and VEGFR/VEGF-A^13^, among others. Due to high intra and inter-patient heterogeneity, the currently available tracers do not cover all PDAC patients^14,15^. Moreover, several of these tracers fail to distinguish between CP and PDAC, or cannot detect micro-metastases^15,16^. In this study, we use a fast and unbiased method to identify tumor targets that are highly expressed in primary tumor tissue compared to surrounding tissue. In addition, we investigate the protein expression of these targets in surrounding lymph nodes to determine whether they could be used to detect lymph node metastasis. Like in several other studies, we have investigated the effect of NAT on target expression^3,4,15^. Currently, potential tumor targets are often discerned based on a literature search on known (PDAC-associated) biomarkers^3,17^. However, this can lead to a biased search in which potential targets may be overlooked^18^. Euretos (Utrecht, The Netherlands) offers a data-driven pre-clinical discovery tool that considers omics data of the Genotype-Tissue Expression (GTEx) and The Cancer Genome Atlas (TCGA)^19^. Its feasibility to identify novel targets has been demonstrated in several recent studies^18,20^. Moreover, this tool enables the differentiation of gene expression levels of targets in tumor tissue, selected healthy tissue, and cell types like immune cells. In this study, we used this data-driven approach to identify PDAC tumor targets that may be suitable for FGS. This search engine specifically visualizes RNA expression levels in selected tissue, rather than protein expression. The protein expression of selected targets was therefore evaluated using immunohistochemistry.

## Results

### PDAC target identification using Euretos

We started by identifying genes that are potential biomarkers for targeting PDAC, based on RNA expression. Using Euretos, we identified membranous targets that are highly expressed in PDAC compared to surrounding tissues, including healthy pancreatic, CP, and duodenal tissues. A gene set consisting of 4673 genes likely to be expressed on the cell surface was used for the data-driven search using the Euretos search engine. After application of the expression thresholds, 11 potential targets remained (Table 1). CEACAM5 and MSLN are well-known and extensively studied PDAC targets^4,14^, and Euretos showed that both had high PDAC TPM rates, along with low TPM rates in healthy pancreatic tissues. As the aim of this study was to investigate lesser-known potential PDAC targets, we decided to include only CEACAM5 as a reference target. MUCL3 was not selected for IHC evaluation due to high expression in selected normal tissue as determined from a preliminary experiment (data not shown). PSCA has been shown to have low specificity and was therefore also excluded^21^. VSIG2 and FXYD3 showed high protein expression in normal tissue (Table 1). We used a cutoff tumor-to-normal (T/N) TPM ratio of 10, as previously reported^10^, to limit the list to a feasible number of targets to be further investigated. Therefore, GJB3 was not selected. Thus, CEACAM5, TMPRSS4, COL17A1, CLDN18, and AQP5 were selected as candidate genes for further investigation.

For effective visualization of PDAC in FGS and clear differentiation from CP, a high expression of tumor targets in epithelial cells combined with low expression in immune cells is ideal, as CP is characterized by extensive immune cell infiltration. The ‘Single Cell Study Viewer’ tool of Euretos was used to further investigate in which types of cells these targets are highly expressed. As shown in Figure S1, the RNA expression levels of the selected genes were all higher in tumor epithelial cells compared to immune cells, including macrophages, mast cells, and natural killer cells.

In addition, a volcano plot analysis was performed to visualize the overlapping genes between the surfaceome gene set and the genes from TCGA that are correlated with PDAC. In this analysis, the focus was solely on the upregulation and downregulation of specific genes in PDAC, without considering healthy pancreas or surrounding tissue. The genes of interest, shown in Table 1, are highlighted in the volcano plot. Each of these genes was significantly upregulated in PDAC (Supplementary Figure S2).

**Table 1:** Characteristics of genes of interest obtained from the Euretos-based search, ranked by T/N ratio in TCGA.

Potential target	Predicted location	T/*N* ratio (TCGA)	TPM PDAC (TCGA)	TPM Panc (TCGA)	PE Panc	PE Duo	PE Liver	3D crystal structure
CEACAM5	Membrane	353.2	257.8	0.7	ND	ND	Low	Yes
MUCL3	Membrane/cytoplasm	152.0	57.8	0.4	ND	ND	ND	No
TMPRSS4	Membrane/intracellular	131.0	72.1	0.6	Medium	Medium	Low	No
COL17A1	Cell junction, desmosome, membrane	96.8	78.4	0.8	ND	ND	ND	No
MSLN	Membrane	67.5	272.7	4.0	ND	ND	ND	No
CLDN18	Membrane	64.4	133.3	2.1	ND	ND	ND	No
PSCA	Membrane	51.8	135.2	2.6	Low	ND	ND	No
AQP5	Membrane, cytoplasmic vesicle membrane	37.9	68.9	1.8	Low	Low	Low	Yes
VSIG2	Membrane	18.9	159.0	8.4	High	High	Medium	No
FXYD3	Membrane	17.4	236.8	13.6	ND	Medium	ND	No
GJB3	Membrane, cell junction	9.3	56.0	6.0	ND	ND	ND	No

The median of the transcripts per million (TPM) values is given. Protein expression (PE) levels are as given by the Human Protein Atlas (HPA), with ND: Not Detected. PDAC: Pancreatic Ductal Adenocarcinoma. Panc: healthy pancreatic tissue. Duo: Duodenal tissue. T/N: PDAC-to-Panc ratio. TCGA: The Cancer Genome Atlas.

### Patient selection and clinicopathological characteristics

Tissue samples were selected from 51 patients who underwent pancreatic resection (Table 2). In the studied cohort, a total of 44 patients were diagnosed with PDAC, and 7 patients were ultimately identified as tumor-free and diagnosed with CP. In the PDAC group, 17 patients received NAT, of whom 12 patients received chemoradiotherapy and 5 patients received chemotherapy. Samples included healthy pancreas tissue from 31 patients, PDAC from 33 patients, Tumor-positive lymph nodes (TPLN) from 26 patients, Tumor-negative lymph nodes (TNLN) from 25 patients, CP from 7 patients, and duodenum from 11 patients.

**Table 2:** Clinicopathological characteristics of PDAC patients (*n* = 44) and CP patients (*n* = 7) in the immunohistochemistry cohort.

Clinical pathological characteristics	Total (*n* = 51)	PDAC (*n* = 44)	PDAC no NAT (*n* = 27)	PDAC NAT (*n* = 17)	CP (*n* = 7)
Age (years), mean (SD)	62.7 (11.1)	64.9 (9.8)	66.7 (9.5)	62.1 (9.8)	49 (9.4)
Gender, n (%)					
Male	26 (51.0)	20 (45.5)	13 (48.1)	7 (41.2)	6 (85.7)
Female	25 (49.0)	24 (54.5)	14 (51.9)	10 (58.8)	1 (14.3)
Surgery type, n (%)					
Pancreaticoduodenectomy	36 (70.6)	33 (75.0)	23 (85.2)	13 (76.5)	3 (42.9)
Pancreatic corpus/tail resection	12 (23.5)	8 (18.2)	2 (7.4)	3 (17.6)	4 (57.1)
Total pancreatectomy	3 (5.9)	3 (6.8)	2 (7.4)	1 (5.9)	0 (0)
Tumor differentiation, n (%)					
Good	–	5 (11.4)	3 (11.1)	1 (5.9)	–
Moderate	–	11 (25.0)	10 (37.0)	1 (5.9)	–
Poor	–	14 (31.8)	11 (40.7)	2 (11.8)	–
Missing	–	14 (31.8)	3 (11.1)	13 (76.5)	–
Primary tumor, n (%)					
pT0	–	2 (4.5)	1 (3.7)	1 (5.9)	–
pT1	–	13 (29.5)	6 (22.2)	7 (41.2)	–
pT2	–	23 (52.3)	15 (55.6)	8 (47.1)	–
pT3	–	6 (13.6))	5 (18.5)	1 (5.9)	–
Regional lymph nodes, n (%)					
pN0	–	11 (25.0)	4 (14.8)	7 (41.2)	–
pN1	–	19 (43.2)	11 (40.7)	8 (47.1)	–
pN2	–	14 (31.8)	12 (44.4)	2 (11.8)	–
NAT, n (%)					
No	34 (66.7)	27 (61.4)	27 (100.0)	0	7 (100.0)
Yes, chemoradiotherapy	12 (23.5)	12 (27.3)	0 (0)	12 (70.6)	0 (0)
Yes, chemotherapy	5 (9.8)	5 (11.4)	0 (0)	5 (29.4)	0 (0)
Tumor size (mm), mean (SD)	–	26.7 (11.5)	30.0 (11.9)	21.6 (8.8)	–

PDAC patients are categorized into two groups: NAT patients and no NAT patients.

*PDAC* pancreatic ductal adenocarcinoma, *CP* chronic pancreatitis, *NAT* neoadjuvant therapy, *SD* standard deviation.

### Evaluation of CEACAM5, TMPRSS4, COL17A1, CLDN18 and AQP5 protein expression

**Fig. 1:**
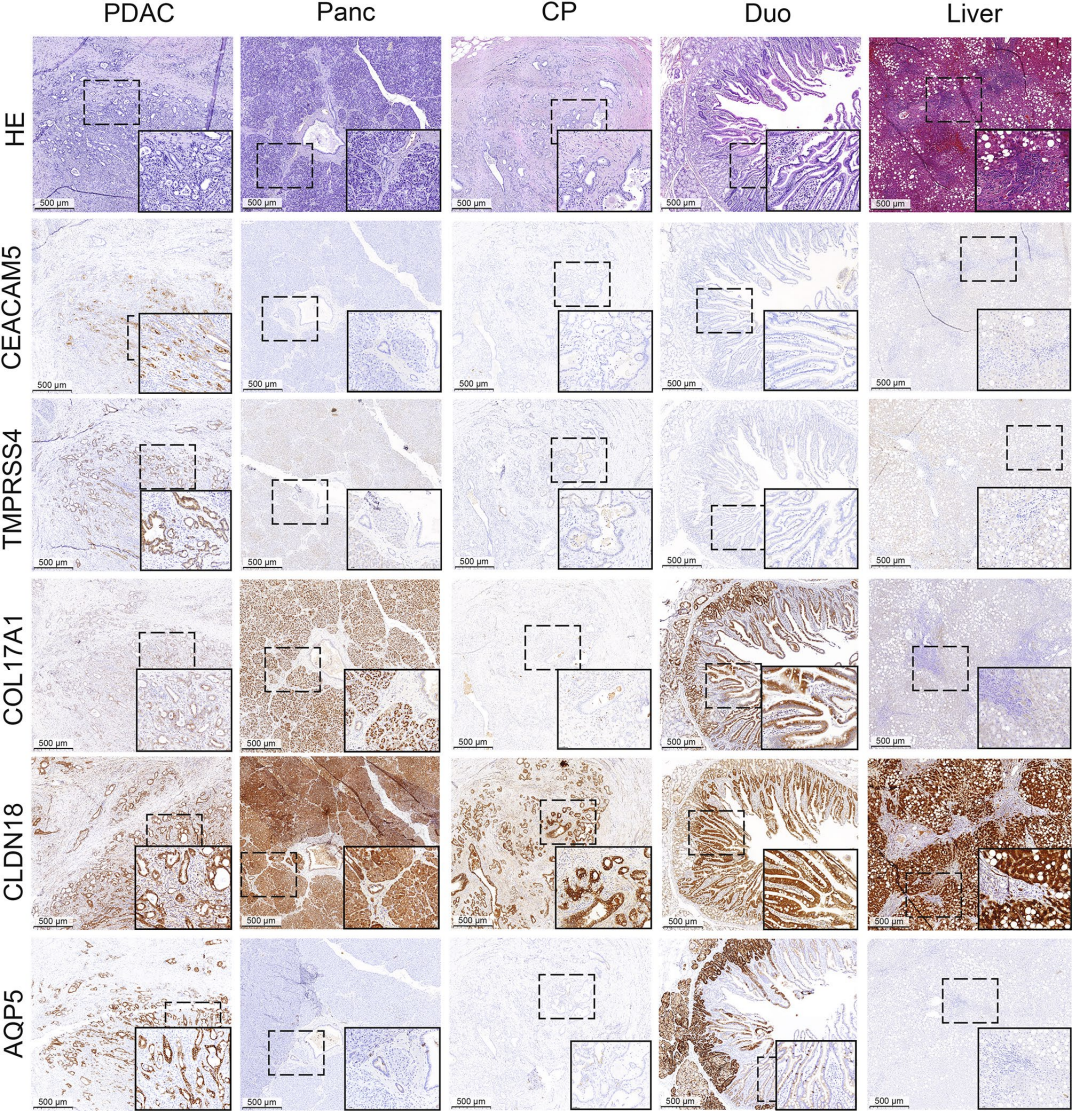
Representative immunohistochemical staining images of tissue sections stained with anti-CEACAM5, anti-TMPRSS4, anti-COL17A1, anti-CLDN18, and anti-AQP5. Scale bars represent 500 μm. HE: hematoxylin and eosin staining. Tissue types: PDAC: pancreatic ductal adenocarcinoma, Panc: pancreas, CP: chronic pancreatitis, Duo: duodenal tissue, Liver: liver tissue.

The protein expressions of CEACAM5, TMPRSS4, COL17A1, CLDN18, and AQP5 in representative PDAC, CP, pancreatic, duodenal, and liver tissue sections are shown in Figure 1.

In PDAC tissue, high levels of CEACAM5 expression were observed, particularly associated with the cell membrane, showing substantial inter-patient variability across the cohort. Protein expression of CEACAM5 was relatively low across healthy pancreatic tissues, including exocrine acinar cells, ducts, and stroma, as well as in duodenal, CP, and liver tissues. TMPRSS4 expression was increased in PDAC tissue, predominantly in epithelial ductal cells, compared to selected healthy tissues, including the pancreas, duodenum, and CP (Fig. 1).

Expression levels of COL17A1 and CLDN18 were moderate and high in PDAC, respectively, and heterogeneous among patients. Moreover, high staining intensity of both proteins was observed in tumor-negative tissues. These proteins were present in pancreatic tissues, specifically within exocrine cells, islets of Langerhans, and epithelial cells lining the ducts. In duodenal tissue, the expression of both targets was predominantly localized to the mucosal layer, with a particular presence in the intestinal glands. In CP tissues, COL17A1 staining showed low intensity, whereas CLDN18 staining marked positive epithelial cells within ducts and regions of fibrotic tissue. In addition, CLDN18 was highly expressed in healthy liver tissue, in which COL17A1 appeared to be minimally expressed.

AQP5 expression was higher in PDAC tissue, particularly in epithelial ductal cells, whereas it remained low in healthy pancreatic, CP, and liver tissues. Notably, AQP5 showed high expression in the intestinal glands of duodenal tissue.

### Protein expression quantification using ImageJ

**Fig. 2:**
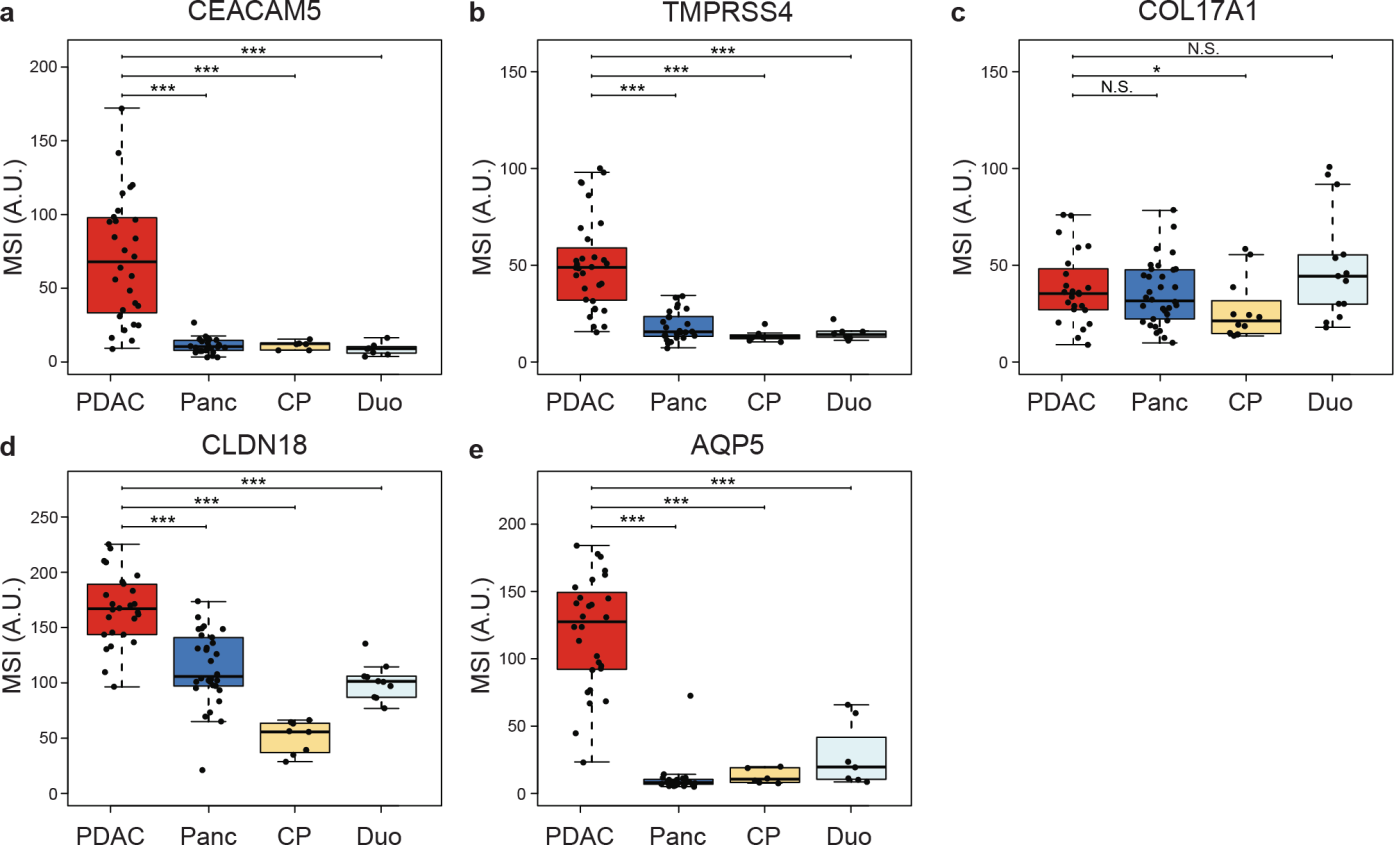
Mean staining intensity (MSI) in tissue sections immunohistochemically stained with anti-CEACAM5, anti-TMPRSS4, anti-COL17A1, anti-CLDN18, and AQP5. (**a**) MSI for CEACAM5 expression. (**b**) MSI for TMPRSS4 expression. (**c**) MSI for COL17A1 expression. (**d**) MSI for CLDN18 expression. (**e**) MSI for AQP5 expression. Thick lines indicate medians, boxes represent the interquartile ranges (IQRs), whiskers indicate the 95% confidence intervals (CI), and circles data from individual patients. Horizontal bars indicate significance levels (Mann-Whitney U-test: ***, *p* < 0.001; **, *p* < 0.01; *, *p* < 0.05; N.S.; not significant). Tissue types: PDAC: pancreatic ductal adenocarcinoma, Panc: pancreas, CP: chronic pancreatitis, DUO: duodenal tissue.

The mean staining intensity (MSI) was quantified for each relevant tissue type (PDAC, pancreas, CP, and duodenum). Figure 2 shows the MSIs of all targets in relevant tissues. For CEACAM5, TMPRSS4, CLDN18, and AQP5, the MSIs in PDAC were significantly higher (all *p* < 0.001) compared to those in pancreatic, CP, and duodenal tissues. For COL17A1, no significant difference was observed between the MSIs of PDAC tissue and pancreatic or duodenal tissues. Expression of COL17A1 was only significantly higher in PDAC compared to CP tissue (*p* < 0.05).

**Fig. 3:**
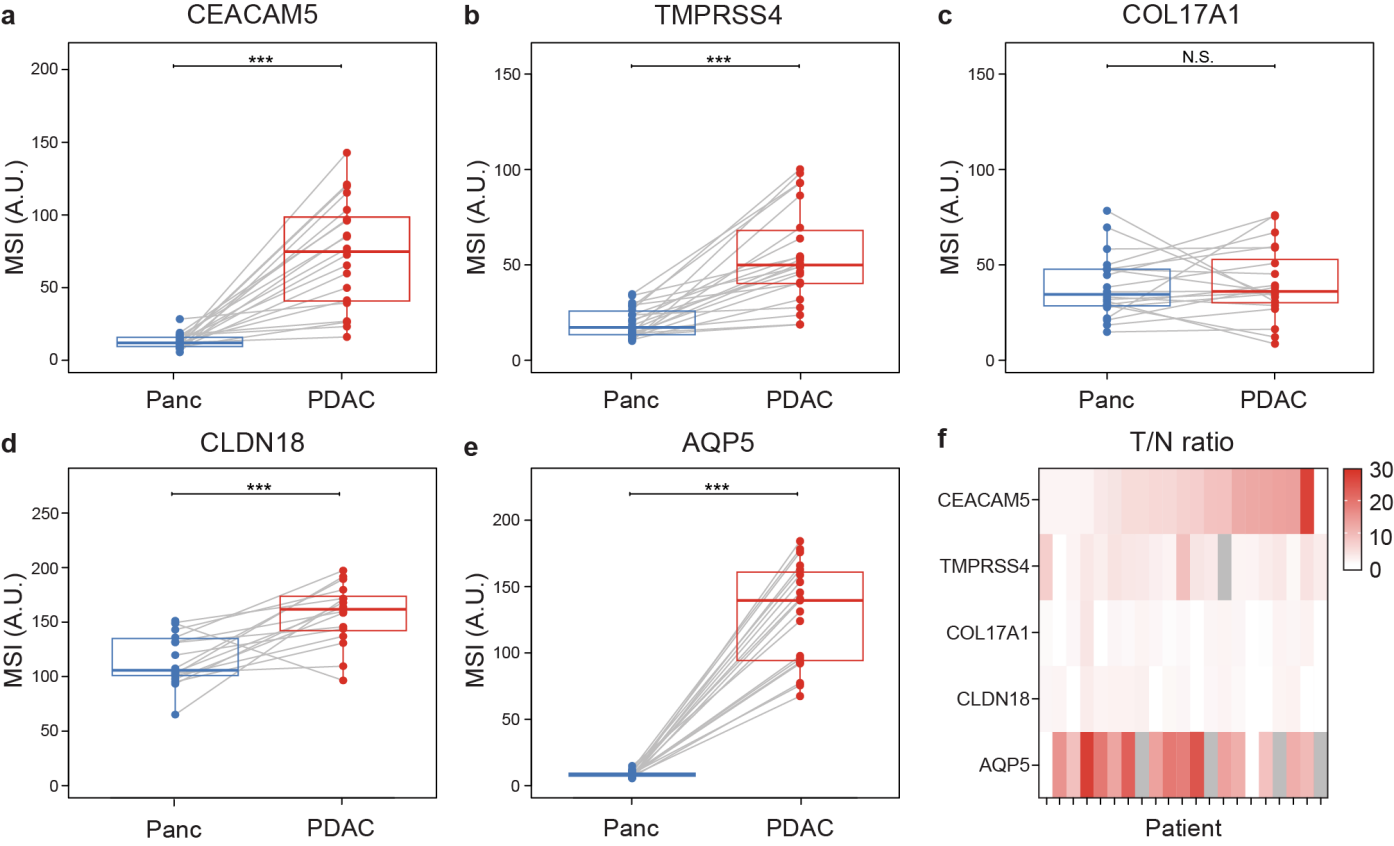
Paired analysis of the mean staining intensities (MSIs) of targets in tissue sections from patients with both pancreatic ductal adenocarcinoma (PDAC) and pancreas (Panc) tissue available. (**a**) MSI for CEACAM5 expression. (**b**) MSI for TMPRSS4 expression. (**c**) MSI for COL17A1 expression. (**d**) MSI for CLDN18 expression. (**e**) MSI for AQP5 expression. Dots and lines represent data from individual patients. Thick lines indicate medians and boxes represent interquartile ranges (IQRs). Horizontal bars indicate significance levels (Wilcoxon-signed rank test: ***, *p* < 0.001; **, *p* < 0.01; *, *p* < 0.05; N.S.; not significant). (**f**) Heat map showing the T/N ratios (MSI PDAC/MSI Panc) for patients with available PDAC and Panc data. Gray regions indicate missing data.

To account for inter-patient heterogeneity in target expression, MSIs of PDAC and relevant tissues were collected and visualized for each patient, as shown in Figure 3. In all patients, CEACAM5, TMPRSS4, and AQP5 showed significantly higher MSIs in PDAC compared to pancreatic tissues (all *p* < 0.001). CLDN18 expression was significantly higher in PDAC tissue compared to healthy pancreatic tissue (*p* < 0.001). However, due to the high average MSI level in pancreatic tissue, the differences in expression were less pronounced in some patients. The difference in MSI between PDAC and pancreatic tissue stained for COL17A1 was not significant. Additionally, COL17A1 expression showed a heterogeneous pattern across patients, with some pancreatic tissues exhibiting higher protein levels than those observed in PDAC tissues.

In Figure 3f, a heat map of T/N ratios for individual patients illustrates the variability of each target across the patient cohort. The mean T/N ratios for CEACAM5, TMPRSS4, COL17A1, CLDN18, and AQP5 were 7.7, 3.2, 1.2, 1.5, and 17.3, respectively. A T/N ratio greater than 10 is considered sufficient for the selection of potential targetable biomarkers for imaging^10^. For CEACAM5 expression, 6 of 19 patients had a T/N ratio greater than 10. In contrast, none of the patients showed a T/N ratio above 10 for TMPRSS4, COL17A1, or CLDN18. However, for AQP5 expression, 16 of 19 patients had a T/N ratio of at least 10. Among the three patients with a T/N ratio below 10 for AQP5, two had T/N ratios higher than those of CEACAM5.

### Target expression after neoadjuvant(chemo)radiotherapy

To determine whether NAT affects tumor target expression levels, MSIs of CEACAM5, TMPRSS4, COL17A1, CLDN18, and AQP5 were compared between patients who received NAT and those who did not receive NAT, as shown in Supplementary Figure S3. Statistical analysis showed no significant differences in MSI values between the NAT and no-NAT groups for any of the investigated target proteins.

### Detection of lymph node metastases with targets of interest

**Fig. 4:**
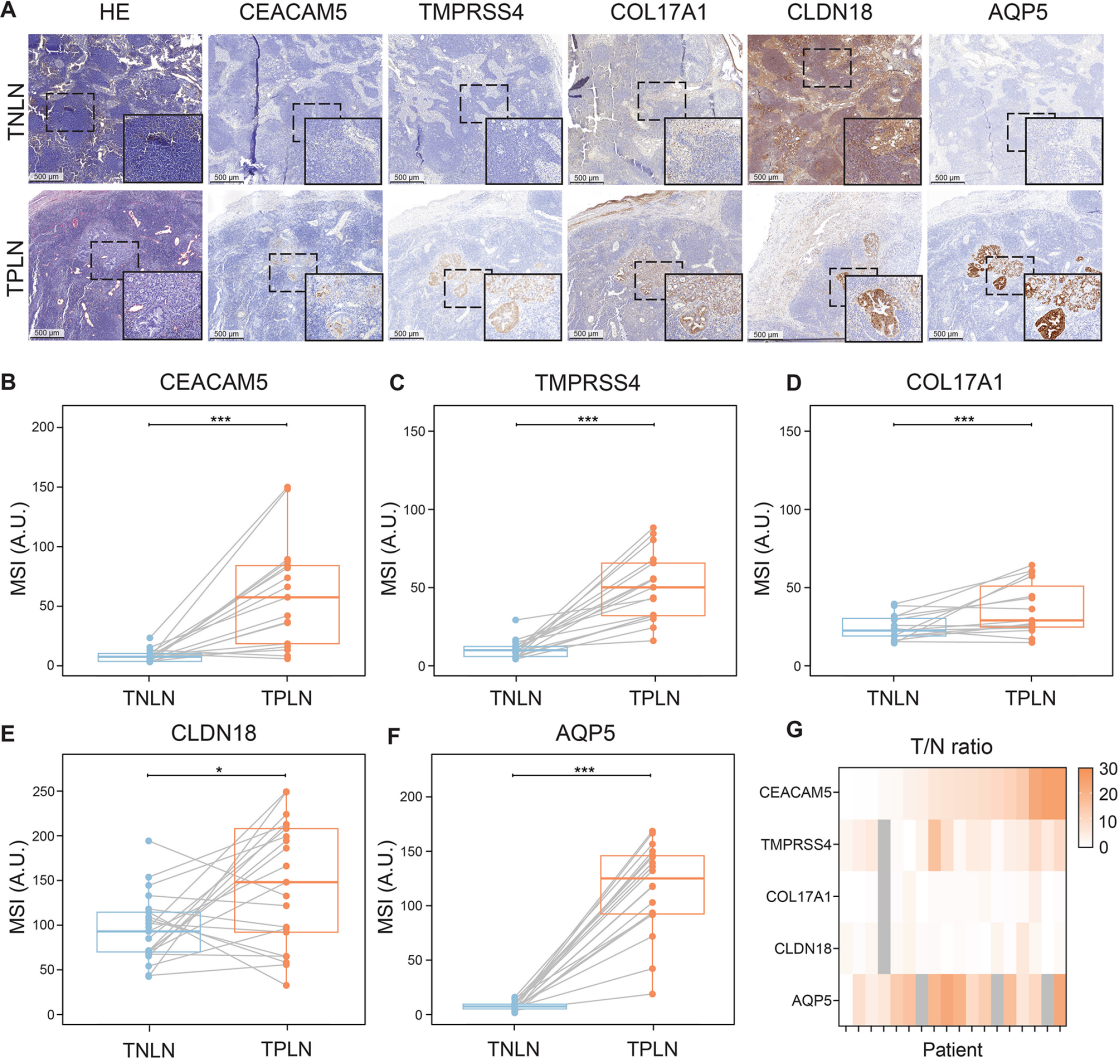
(**a**) Representative immunohistochemical (IHC) staining of tissue sections stained with anti-CEACAM5, anti-TMPRSS4, anti-COL17A1, anti-CLDN18, and anti-AQP5. Scale bars represent 500 µm. HE: hematoxylin and eosin staining. Tissue types: TNLN: Tumor-negative lymph node, TPLN: Tumor-positive lymph node. (**b**–**f**) Paired analysis of the mean staining intensities (MSIs) of targets in tissue sections from patients with both TNLN and TPLN tissues available. (**b**) MSI for CEACAM5 expression. (**c**) MSI for TMPRSS4 expression. (**d**) MSI for COL17A1 expression. (**e**) MSI for CLDN18 expression. (**f**) MSI for AQP5 expression. Dots and lines represent data of individual patients. Thick lines indicate medians and boxes represent interquartile ranges (IQRs). Horizontal bars indicate significance levels (Wilcoxon-signed rank test: ***, *p* < 0.001; **, *p* < 0.01; *, *p* < 0.05; N.S. = not significant). (**g**) Heat map showing the T/N ratios (MSI TPLN / MSI TNLN) for patients in whom TNLN and TPLN data were available. Gray regions indicate missing data.

The potential of the selected targets in detecting lymph node metastases was evaluated by immunohistochemical staining of TPLN and TNLN tissues from the same patients (Fig. 4). As shown in Figure 4a, high levels of CEACAM5 expression were observed in TPLN within PDAC epithelial cells, whereas CEACAM5 expression was low in TNLN. A similar pattern was observed for TMPRSS4, and COL17A1 showed this to a lesser extent. Conversely, CLDN18 had higher expression levels in TNLN compared to TPLN. Lastly, AQP5 expression was low in TNLN and high in TPLN, as shown in Figure 4a. Staining intensities of all targets were significantly higher in TPLN tissue (all *p* < 0.001, except for CLDN18 for which *p* < 0.05) compared to TNLN tissue as illustrated in Figures 4b-f.

In addition, a heat map of TPLN/TNLN T/N ratios for individual patients (Fig. 4g) shows the heterogeneity of each target across the patient population. The mean T/N ratio for CEACAM5, TMPRSS4, COL17A1, CLDN18, and AQP5 is 9.7, 5.9, 1.6, 1.8, and 9.7, respectively. For CEACAM5 expression, 6 of 17 patients had a T/N ratio above 10. TMPRSS4 showed this level of expression in 1 of 16 patients, while none of the patients exceeded this threshold for COL17A1 and CLDN18. In contrast, AQP5 protein expression was increased in the TPLN tissue of 13 of 18 patients, with T/N ratios reaching at least 10.

## Discussion

FGS is useful when tumor identification is limited. Untargeted moieties, e.g., ICG, are recognized to lack the specificity to guide surgeons in pancreatic cancer and therefore achieve complete tumor and metastatic lymph node resection^8^. In this study, a data-driven approach was used to identify upregulated genes encoding for cell-surface proteins that can be targeted during FGS of PDAC. CEACAM5, TMPRSS4, COL17A1, CLDN18, and AQP5 were selected for further evaluation based on their RNA expression and predicted protein expression. Analysis of IHC stains revealed high protein expression levels of CEACAM5, TMPRSS4, and AQP5 in PDAC compared to healthy pancreatic tissue. Moreover, these tumor targets were significantly higher expressed in PDAC than in CP, supporting their use as intraoperative biomarkers to differentiate PDAC from CP. Although COL17A1 and CLDN18 were initially selected as potential targets based on their RNA expression and predicted protein levels, high protein expression was also detected in normal healthy pancreatic tissue. This highlights the importance of evaluating protein expression to refine target selection. The study also investigated several other factors relevant to potential FGS targets for pancreatic cancer. To assess the suitability of these targets for distinguishing locally advanced pancreatic head carcinomas with duodenal invasion^15^, protein expression was also evaluated in healthy duodenal tissue. CEACAM5, TMPRSS4, CLDN18, and AQP5 expressions were significantly higher in PDAC than in duodenal tissue. Moreover, tumor target expression remained consistent in stains of both NAT-treated and untreated patients for all targets, suggesting that neoadjuvant therapy does not affect target expression in PDAC tumors. Furthermore, CEACAM5 and AQP5 were significantly higher expressed in tumor-positive compared to tumor-negative lymph nodes and demonstrated particularly high T/N ratios, indicating their potential to detect metastatic lymph nodes for FGS and staging^15^.

Our target selection was based on a data-driven search in Euretos. Among the top selected genes of interest, CEACAM5, PSCA, and MSLN were also included, all of which are already well-studied targets for the treatment and imaging of PDAC^4,14,22,23^. The IHC staining results of CEACAM5 showed high protein expression in PDAC but with substantial inter-patient heterogeneity. Heterogeneity of CEACAM5 expression has been observed before and may be associated with neoadjuvant therapy^3,4^. In our study, the protein expression of the selected targets, including CEACAM5, was similar between patients who received NAT and those who did not. However, our sample size for these particular analyses was relatively limited, which may have affected the results. TMPRSS4 protein expression in PDAC has been studied previously and has been identified as a promising therapeutic target^24^. Similar IHC staining patterns observed in this study further support its potential applicability for FGS. COL17A1, in contrast to previous IHC findings^25^, was not found to be a suitable target due to its high protein expression in both PDAC and healthy pancreatic tissues. CLDN18 has previously been identified as a biomarker for PDAC using a data-driven approach^26^. The utility of CLDN18 for image-guided surgery has been described and two CLDN18-specific imaging probes were able to facilitate the resection of tumor tissue in orthotopic mouse tumor models^27^. We observed high protein expression levels of CLDN18 in normal pancreatic tissues, adding to conflicting findings reported in the literature about CLDN18 expression^28,29^. The conflicting findings reflect the broader inconsistency in the literature regarding protein expression patterns of COL17A1 and CLDN18. These differences may partially result from variability in the affinity of the antibodies or epitope recognition. As such, future studies employing multiple antibodies per target could provide additional clarity and aid in distinguishing between technical variation and biological differences.

AQP5 and COL17A1 expression have recently been linked to PDAC progression^25,30^. In addition, AQP5 expression has been related to PDAC tumor differentiation^31^. In our study, AQP5 T/N ratios were consistently high over all patients relative to the other studied targets. AQP5 did exhibit expression in normal ductal epithelial cells, consistent with previous research^32^. The absence in the top-ranked results of other widely investigated targets, such as EGFR, can be attributed to their relatively high or ambiguously reported expression levels in healthy pancreatic tissues^33^. This led to their exclusion during the selection process. It must be noted that the found T/N ratios may not reflect clinically feasible T/N ratios as they are dependent on conditions of IHC, for instance, dilution and the affinity of the used antibody to the target. In addition, some already-known targets have T/N ratios of less than 10 and are used as biomarkers for FGS^10^.

Traditional target screening methods, including literature searches, may be subject to several kinds of bias, such as publication bias. By combining omics data with artificial intelligence to handle various data inputs, data-driven tools facilitate the unbiased discovery of therapeutic targets that traditional techniques may fail to detect^34^. In this study, we used Euretos as its potential had been demonstrated previously^18,20^. Other data-driven approaches have been shown to be successful in identifying PDAC targets as well^26,35,36^. However, a limitation of the use of bulk RNA sequencing data is that samples contain a mixture of different cell types, which can be misleading when a biomarker is only present in a subset of cells^37^. Single-cell RNA sequencing overcomes this limitation by analyzing expression at the individual cell level. Euretos’ ‘Single Cell Study Viewer’ tool was used in this study to ensure that promising targets were also all highly expressed in epithelial cells and minimally in immune cells. An additional limitation may be the predominant use of TCGA data, which mainly includes cancer and less healthy tissue samples, and may be subject to batch effects or other biases^35^. In this study, GTEx data were not included as Euretos does not currently apply a normalization strategy compatible with combining GTEx and TCGA datasets. Integrating these datasets could provide valuable insights by enabling more accurate comparison between normal and tumor tissue expression^38^. Moreover, some potential targets could not be retrieved in Euretos. Of one known target, αvβ6^3,33,39^, only the gene encoding for the subunit B6 (ITGB6) could be found. Prior research indicates a relatively high expression of this gene in normal tissue^3,4^. In addition, Euretos may not recognize potential targets that originate from the same gene but have different isoforms. For instance, although CD44 has multiple isoforms, the CD44 variant 6 (v6) has been specifically identified and used as a target for FGS in head and neck squamous cell carcinoma^40^. Nevertheless, a combination of search strategies, e.g. adding literature screening, could aid the selection of targets^20^. Lastly, one of the major limitations of the use of omics search tools for tumor target identification is that RNA expression does not always correlate with protein expression^41^, as was shown for COL17A1 and CLDN18. Post-transcriptional modifications can affect the stability, location, and translation efficiency. Moreover, protein turnover dynamics determine the efficiency of the complex networks in which proteins function. This was addressed by evaluating protein expression through IHC, which is a key strength of this research. Data-driven approaches specifically focusing on protein expression and taking into account isoforms or multimers would help identify novel targets.

A key challenge in tumor target identification is to find targets that are consistently highly expressed across all patients and within patients. In this study, we focused on inter-patient heterogeneity, showing that target expression in PDAC is subject to substantial variability between patients, as previously indicated^14^. Euretos helps address this challenge by enabling the user to visualize patient-specific data. For future research, it will be important to also consider intra-patient heterogeneity, particularly when translating PDAC tumor targets into clinical applications. Developing FGS probes that are robust to inter- and intra-patient heterogeneity, including multi-target tracers, may enhance the effectiveness of PDAC resection surgeries^33^. However, it may be challenging to design a tracer that can simultaneously bind to multiple targets with high affinity. Another interesting development to handle target heterogeneity is multiplex imaging, that is, injecting an array of probes targeting several tumor biomarkers and potentially using multiple imaging modalities^42^. Based on our study’s findings, imaging of tumors using AQP5 combined with CEACAM5 and/or TMPRSS4 may enhance the detection of PDAC across a broader patient population compared to any of the targets alone. The availability of the protein crystal structures of AQP5 and CEACAM5 also makes these promising candidates for further probe development using computational screening programs^39^.

The data-driven selection method using Euretos proved to be a valuable tool for identifying imaging targets in pancreatic cancer. While experimental validation of protein expression is still necessary, this approach allows for rapid and unbiased selection of potential tumor targets for FGS. Four of the five candidate targets selected showed significantly higher expression in PDAC than surrounding tissues and could detect lymph node metastases. Among the potential targets for FGS, AQP5 emerged as the most promising, alongside CEACAM5 and TMPRSS4, due to its especially low expression in healthy tissue. A combination of AQP5 with CEACAM5 and/or TMPRSS4 holds the most promise for addressing the interpatient heterogeneity of PDAC.

## Methods

### Patient and tissue selection

Formalin-fixed, paraffin-embedded (FFPE) tissue blocks from 51 patients who underwent pancreatic resection between 2011 and 2020 at the Leiden University Medical Centre (LUMC) and were diagnosed with PDAC or CP were selected for this study. NAT was administered to 17 of these patients. The tissue blocks contained PDAC, Panc, CP, TPLN, TNLN, and/or duodenal tissue. Medical and pathology records were retrospectively collected. This study was approved by the Gastroenterology Biobank Review Committee (protocol reference: 2020-16), and the local medical ethics review committee (protocol reference: B20.052). The study was conducted in compliance with the Dutch code of conduct for the responsible use of human tissue in medical research.

### Biomarker selection

Using the Euretos search engine (accessed in May 2023; Utrecht, The Netherlands), we compared mRNA levels in transcripts per million (TPM) in Pancreatic Ductal Adenocarcinoma (PDAC) tissue with healthy tissue, obtained from TCGA. The surfaceome gene set was used to select genes that are predicted to encode proteins expressed on the cell surface. This gene set is based on four sources, including two computational surfaceome lists, mass-spectrometry data obtained from Cell Surface Protein Atlas (CSPA), and an Euretos surfaceome list^43–46^. Expression thresholds were established for normal pancreas tissue (< 15 TPM), surrounding tissue (bile duct and liver, < 30 TPM), and PDAC (> 50 TPM). The tumor-to-normal (T/N) ratio calculation for the ranking of genes was performed using the TCGA dataset exclusively. We sorted the resulting targets by their T/N ratio. In addition, the selection process took into account the following criteria: (1) expression in normal tissues should not be higher than 50 TPM, (2) protein should be expressed on the membrane according to UniProt and the Human Protein Atlas (HPA), (3) protein expression in normal tissue according to the HPA should be low, and (4) a 3D crystal structure or predicted AlphaFold 2 (DeepMind, London, UK) structure with high confidence should be available to enable computational probe development. The gene set was additionally validated through the utilization of the ‘Single Cell Study Viewer’ tool in Euretos^47^. This tool allows the estimation of expression levels per cell type in PDAC patients. To illustrate the RNA expression of the selected targets, a volcano plot with Log_2_-fold changes was created using VolcaNoseR^48^. The input data consisted of genes from the surfaceome gene set and genes from TCGA associated with PDAC, filtered using the Xena Functional Genomics Explorer (University of California, Santa Cruz). cBioPortal was used to filter gene data specifically from PDAC patients.

### Hematoxylin and Eosin (HE) and immunohistochemistry (IHC) staining

FFPE tissue blocks were cut into sections of 4 μm thickness. For hematoxylin and eosin staining, slides were twice incubated in xylene for 5 min, then rehydrated through a graded ethanol series (100%, 70%, 50%) and demineralized water. Sections were stained in Mayer’s Hematoxylin (Klinipath, Duiven, The Netherlands) for 4 min, rinsed in tap water (10 min) and demineralized water (1 min), and then incubated in eosin (Dako, Glostrup, Denmark) for 2 min. After thorough rinsing in demineralized water, slides were dehydrated with ethanol concentrations of 50%, 70%, and 100% respectively. Lastly, the slides were incubated twice in xylene for 5 min and mounted in Pertex (Histolab, Askim, Sweden).

For immunohistochemical staining, tissue sections were deparaffinized in a series of xylene and rehydrated in descending ethanol solutions of 100%, 70%, and 50%. After washing with demineralized water, endogenous peroxidase was blocked with 0.3% hydrogen peroxide (Merck, New Jersey, USA) for 20 min. Depending on the antibody of interest, different antigen retrieval conditions were used in EnVision Flex Target Retrieval Solution using the PT-Link module (Dako, Glostrup, Denmark). The antigen retrieval consisted of heating at 95 °C for 10 min with different pH levels, which are shown in supplementary Table S1. After cooling and washing three times with phosphate-buffered saline (PBS), the slides were incubated with a solution of primary antibody in PBS with 1% BSA. The primary antibody concentrations and characteristics can be found in Supplementary Table S1. The incubation step took place in a humidified closed chamber at room temperature (RT) overnight. In the specific case of TMPRSS4, a blocking step was performed using 5% normal goat serum for 10 min before the incubation with the primary antibodies. Next, the slides were washed three times with PBS and incubated with horseradish peroxidase (HRP)-labeled secondary antibody (anti-rabbit or anti-mouse Envision, Dako) for 30 min at RT. After washing three times with PBS, the staining was visualized using 3,3’Diaminobenzidine (DAB) substrate buffer (Dako) for 10 min. Afterwards, DAB was deactivated by washing twice with demineralized water and counterstained with Mayer’s Hematoxylin for 15 s. Finally, the slides were rinsed with tap water for 5 min, dehydrated at 37 °C for one hour, and mounted in Pertex.

### IHC staining scoring: semi-automated imaging analysis

The slides were scanned using the PANNORAMIC 250 Flash III scanner (3DHISTECH, Budapest, Hungary) at 20x magnification. Multiple regions of interest (ROIs) of PDAC, pancreatic, TPLN, TNLN, and/or duodenal tissues were selected per patient. In healthy pancreatic tissue, representative regions were selected that included exocrine cells, ducts, islets of Langerhans, and other pancreatic structures. In PDAC samples, only tumor ductal epithelial cells were selected as ROIs to assess target protein expression specifically within the malignant cell population. The regions were annotated by an experienced hepato-pancreato-biliary pathologist (A.S.L.P.C.). The mean staining intensity (MSI) of an annotated region was determined using ImageJ (1.54d, Java 1.8.0_345).

### Statistical analysis

Statistical analysis was performed using RStudio (4.2.1). Wilcoxon signed-rank tests and Mann-Whitney U tests were used to compare paired and unpaired data, respectively, with a significance treshold of  α = 0.05.

## Electronic supplementary material

Below is the link to the electronic supplementary material.

Supplementary Material 1

## Data Availability

The datasets generated and analyzed during the current study are available from the corresponding author upon reasonable request.

## Abbreviations

CP: Chronic pancreatitis
CSPA: Cell-surface protein atlas
DAB: 3,3’-diaminobenzidine
Duo: Duodenal tissue
FFPE: Formalin-fixed paraffin-embedded
FGS: Fluorescence-guided surgery
GTex: Genotype-tissue expression
HE: Hematoxylin and eosin
HPA: Human Protein Atlas
HRP: Horseradish peroxidase
ICG: Indocyanine green
IHC: Immunohistochemistry
MSI: Mean staining intensity
NAT: Neoadjuvant therapy
NIR: Near-infrared
Panc: Pancreatic tissue
PBS: Phosphate-buffered saline
PDAC: Pancreatic ductal adenocarcinoma
PE: Protein expression
R0: Complete resections
R1: Incomplete resections
ROI: Regions of interest
RT: Room temperature
TCGA: The Cancer Genome Atlas
TNLN: Tumor-negative lymph node
T/N: Tumor-to-normal ratio
TPLN: Tumor-positive lymph node
TPM: Transcript per million
