# Supplementary material for: Evaluation of tumor targets selected from public genomic databases for imaging of pancreatic ductal adenocarcinoma
Source: Sci Rep. 2025 May 16;15:17102. doi: 10.1038/s41598-025-00517-1 (PMC12084321; doi:10.1038/s41598-025-00517-1)
Supplement: Supplementary file 1 — Supplementary Material 1 [file 41598_2025_517_MOESM1_ESM.docx]

**Supplementary File**

***Table S1****.* *Overview and characteristics of used antibodies during IHC staining. Optimal dilutions and antigen retrieval of the antibodies were selected based on multiple titrations in PDAC and tissues with high protein expression of the specific targets.*

| **Antibody** | **Manufacturer** | **Species** | **Article nr.** | **Lot nr.** | **Concentration (mg/mL)** | **Dilution** | **Antigen retrieval** |
| --- | --- | --- | --- | --- | --- | --- | --- |
| anti-CEACAM5 | Santa Cruz | Mouse,  monoclonal | Sc-23928 | K1914 | 0.50 | 1:1000 | pH 6.0, citrate buffer (Dako) |
| anti-AQP5 | Proteintech | Rabbit,  polyclonal | 11283-I-AP | 00048108 | 0.30 | 1:1600 | pH 6.0, citrate buffer (Dako) |
| anti-CLDN18 | Invitrogen | Rabbit,  monoclonal | AB_2532290 | 2503093 | 0.50 | 1:2000 | pH 9.0. Tris-EDTA (Dako) |
| anti-TMPRSS4 | Abcam | Rabbit,  monoclonal | Ab92320 | GR3273694-6 | 0.33 | 1:2400 | pH 9.0, Tris-EDTA (Dako) |
| anti-COL17A1 | Sigma-Aldrich | Rabbit,  polyclonal | HPA052963 | 26158 | 0.05 | 1:200 | pH 9.0, Tris-EDTA (Dako) |
|  |  |  |  |  |  |  |  |

**Results of the Single Cell Study Viewer**

For effective visualizing PDAC in FGS, high expression of tumor targets in epithelial cells and low expression in immune cells is preferred. This differentiation is necessary because chronic pancreatitis (CP) is associated with extensive immune cell infiltration, unlike PDAC. The Single Cell Study viewer offers a comprehensive overview of single-cell RNA sequencing (RNAseq) data from a study by Steele et al., published in Nature Cancer in 2020. The dataset includes samples from four healthy individuals and sixteen patients with pancreatic ductal adenocarcinoma (PDAC). This tool facilitates the evaluation of candidate target expression in specific cell types. As shown in Figure S1, all genes had higher expression levels in epithelial cells than immune cells including macrophages, mast cells, and natural killer cells.


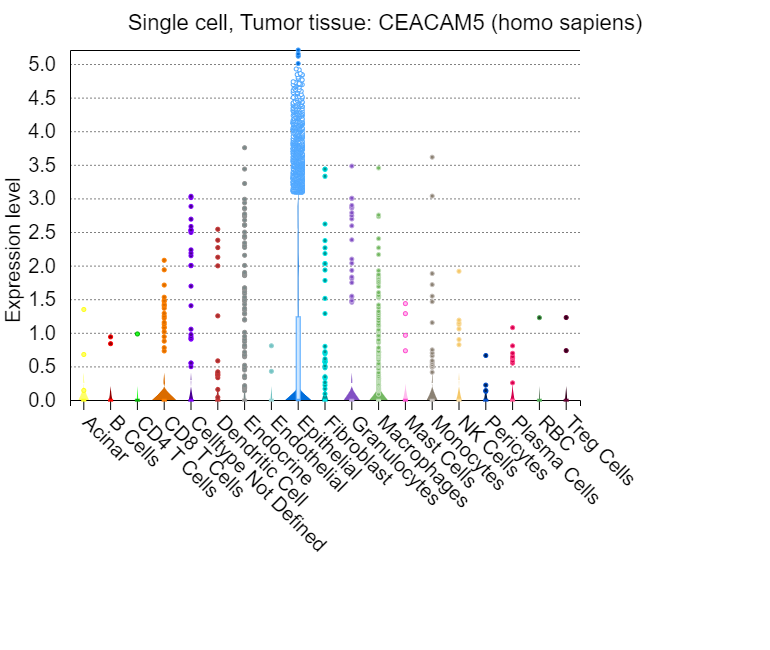

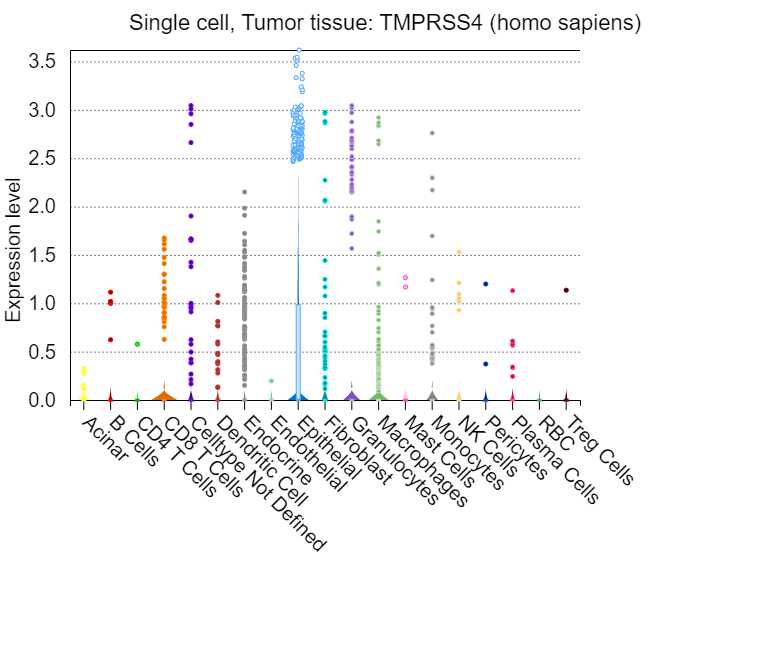


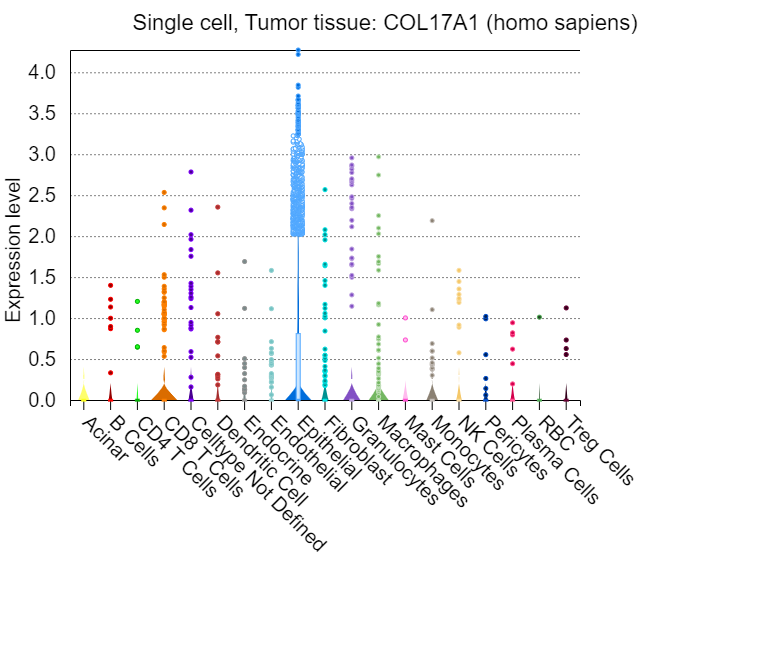

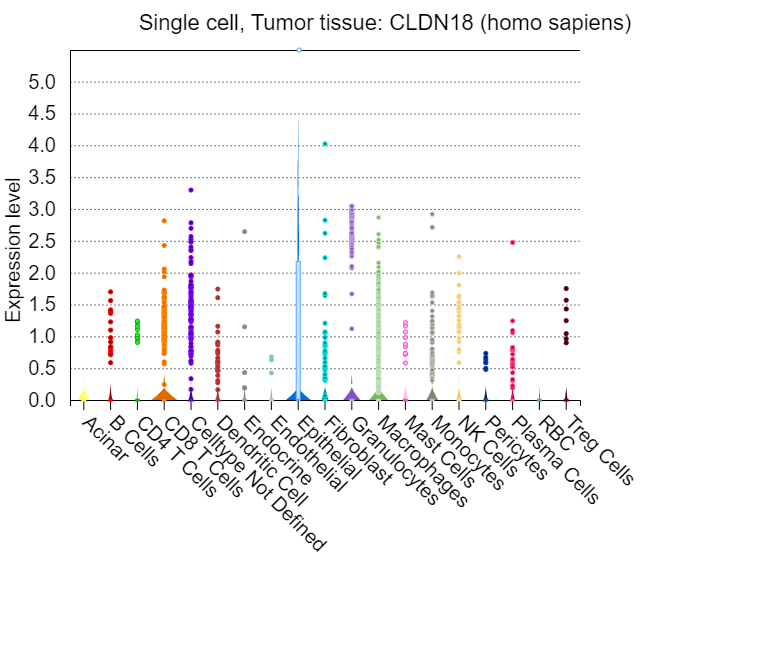


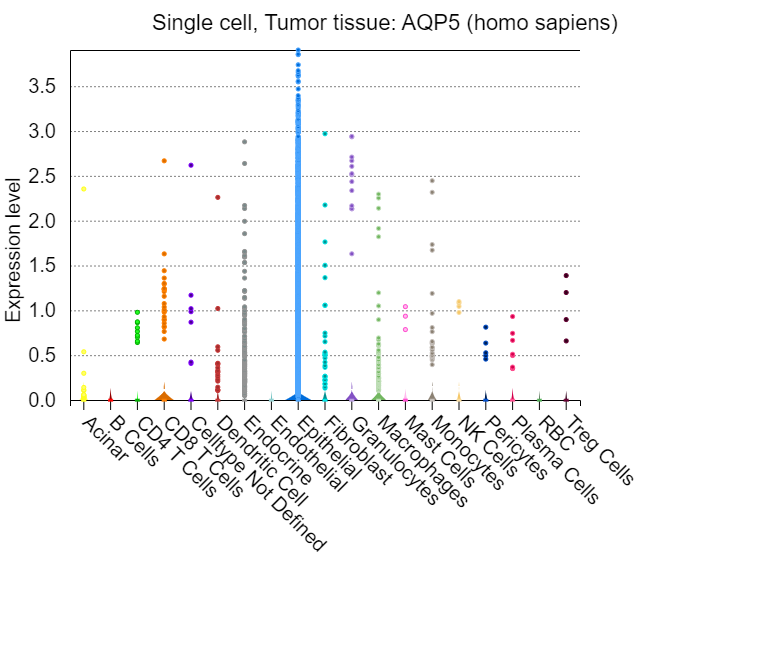


***Figure S1:*** *Overview of plots derived from the single-cell study for the selected five candidate targets: CEACAM5, TMPRSS4, COL17A1, CLDN18, and AQP5. The raw counts of genes in each human cell were first normalized by dividing by the total gene counts in that cell. This normalized value is then scaled by multiplying by 10,000. Finally, the scaled value undergoes a log transformation using log1p for further analysis.*

*
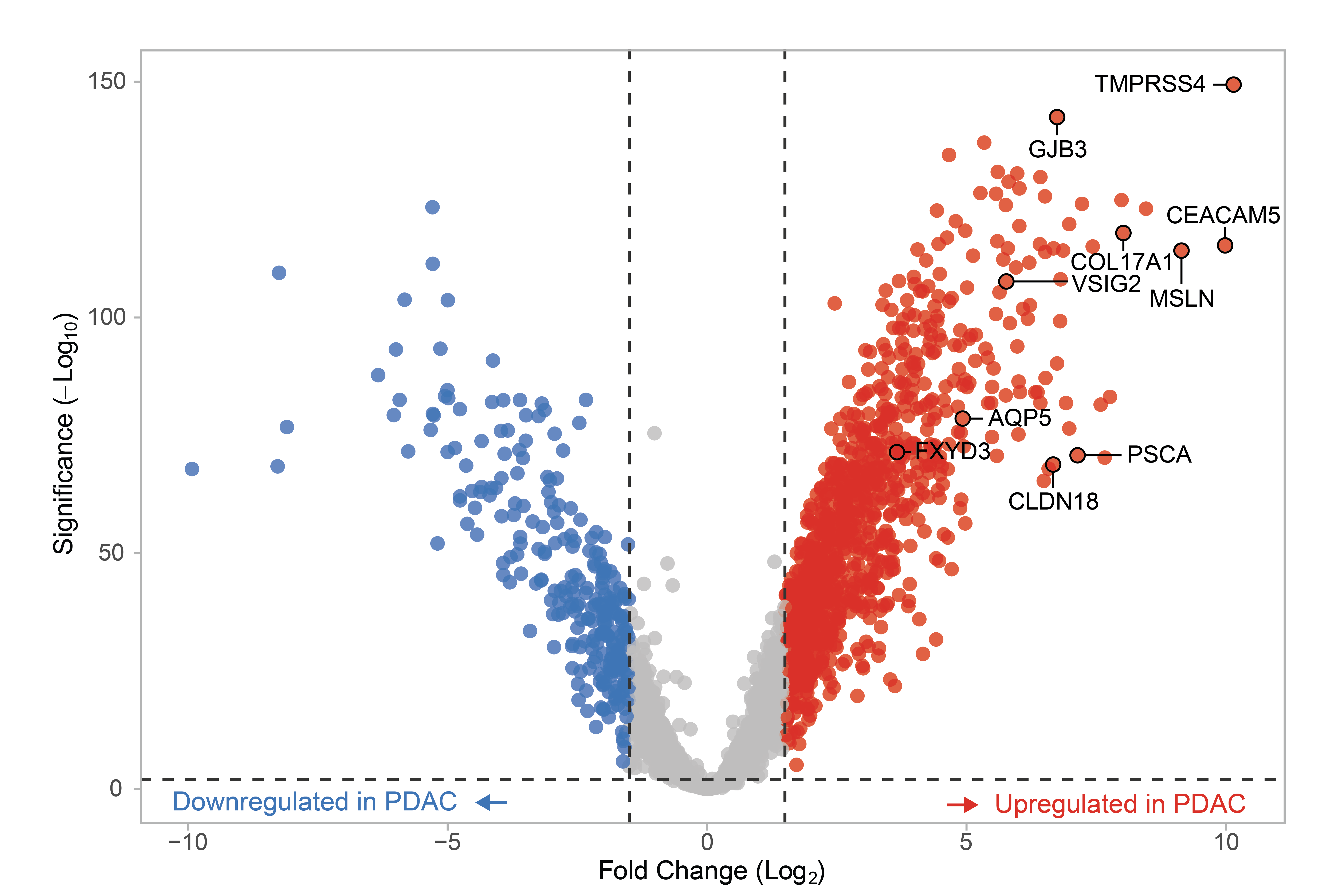
*

***Figure S2****. A volcano plot consisting of overlapping genes of the surfaceome gene set and genes from TCGA that are associated with PDAC. The top genes obtained from the Euretos search are indicated in the plot. The y-axis of the plot represents the negative logarithm (-log) of the level of significance, while the x-axis represents the Log2-fold change. Genes depicted in red indicate upregulation in PDAC, whereas genes depicted in blue indicate downregulation.*

***
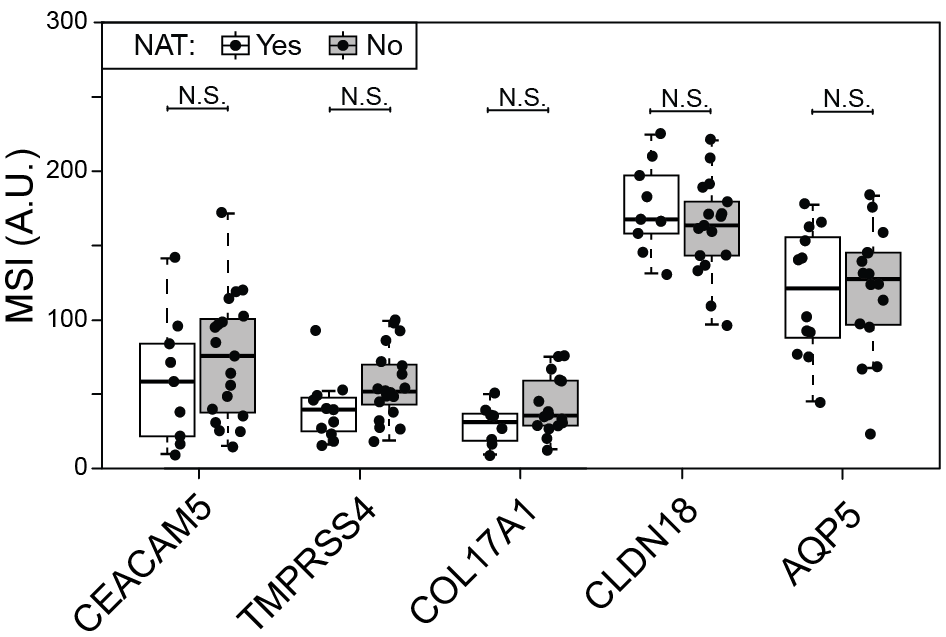
***

***Figure S3.*** *Mean staining intensity (MSI) in PDAC tissue sections stained with anti-CEACAM5, anti-TMPRSS4, anti-COL17A1, anti-CLDN18, and anti-AQP5 of patients who received neoadjuvant therapy (NAT) or no NAT. Thick lines indicate medians, boxes interquartile range (IQR), whiskers 95% confidence intervals (CI), and circles data of individual patients. Horizontal bars indicate significance levels (Mann-Whitney U-test: ***, p < 0.001; **, p < 0.01; *, p < 0.05; N.S. = not significant).*
